# Sex-specific effects of dietary fatty acids on saliva cortisol and social behavior in guinea pigs under different social environmental conditions

**DOI:** 10.1186/s13293-016-0107-5

**Published:** 2016-09-22

**Authors:** Matthias Nemeth, Eva Millesi, Verena Puehringer-Sturmayr, Arthur Kaplan, Karl-Heinz Wagner, Ruth Quint, Bernard Wallner

**Affiliations:** 1 Department of Behavioural Biology, University of Vienna, Althanstrasse 14, 1090 Vienna, Austria; 2 Department of Nutritional Sciences, University of Vienna, Althanstrasse 14, 1090 Vienna, Austria; 3 Department of Anthropology, University of Vienna, Althanstrasse 14, 1090 Vienna, Austria

**Keywords:** Polyunsaturated fatty acid, Saturated fatty acid, Saliva cortisol concentrations, Social confrontations, Social behavior, Social hierarchy

## Abstract

**Background:**

Unbalanced dietary intakes of saturated (SFAs) and polyunsaturated (PUFAs) fatty acids can profoundly influence the hypothalamic-pituitary-adrenal (HPA)-axis and glucocorticoid secretions in relation to behavioral performances. The beneficial effects of higher dietary PUFA intakes and PUFA:SFA ratios may also affect social interactions and social-living per se, where adequate physiological and behavioral responses are essential to cope with unstable social environmental conditions.

**Methods:**

Effects of diets high in PUFAs or SFAs and a control diet were investigated in male and female guinea pigs after 60 days of supplementation. Plasma fatty acid patterns served as an indicator of the general fatty acid status. HPA-axis activities, determined by measuring saliva cortisol concentrations, social behaviors, and hierarchy ranks were analyzed during group housing of established single-sexed groups and during challenging social confrontations with unfamiliar individuals of the other groups.

**Results:**

The plasma PUFA:SFA ratio was highest in PUFA supplemented animals, with female levels significantly exceeding males, and lowest in SFA animals. SFA males and females showed increased saliva cortisol levels and decreased aggressiveness during group housing, while sociopositive behaviors were lowest in PUFA males. Males generally showed higher cortisol increases in response to the challenging social confrontations with unfamiliar individuals than females. While increasing cortisol concentrations were detected in control and PUFA animals, no such effect was found in SFA animals. During social confrontations, PUFA males showed higher levels of agonistic and sociopositive behaviors and also gained higher dominance ranks among males, which was not detected for females.

**Conclusions:**

While SFAs seemingly impaired cortisol responses and social behaviors, PUFAs enabled adequate behavioral responses in male individuals under stressful new social environmental conditions. This sex-specific effect was possibly related to a general sex difference in the n-3 PUFA bioavailability and cortisol responses, which may indicate that males are more susceptible to changing environmental conditions, and shows how dietary fatty acids can shape social systems.

**Electronic supplementary material:**

The online version of this article (doi:10.1186/s13293-016-0107-5) contains supplementary material, which is available to authorized users.

## Background

The social environment plays an essential role in the modulation of the hypothalamic-pituitary-adrenal (HPA)-axis in relation to glucocorticoid secretion rates [1, 2]. Glucocorticoids such as cortisol, released by the adrenal glands, show a wide range of physiological actions and are involved in the adjustment of behavioral and physiological functions to different stressful events. An adequate HPA-axis response to unpredictable and challenging social environmental conditions, which is usually characterized by elevated glucocorticoid secretion rates, is therefore of major importance to cope with such stressors [3–5]. HPA-axis dysfunctions, in contrast, can impair behavioral responses and even evoke brain- and metabolic-related diseases [6]. In this context, it has been suggested that balanced dietary intakes of saturated (SFAs) and polyunsaturated (PUFAs) fatty acids and their consequent availability for metabolic processes and integration into neuronal cell membranes can modulate physiological and behavioral functions [7, 8].

The essential omega-3 (n-3) and omega-6 (n-6) PUFAs alpha-linolenic acid (ALA, 18:3 n-3) and linoleic acid (LA, 18:2 n-6), which have to be ingested with the diet, and their long-chain metabolites eicosapentaenoic acid (EPA, 20:5 n-3), docosahexaenoic acid (DHA, 22:6 n-3), and arachidonic acid (AA, 20:4 n-6) are of tremendous biological relevance [9]. Considering the general benefits of lower dietary n-6:n-3 ratios for neurophysiological processes [10, 11], PUFAs can positively affect a variety of brain-related behavioral functions [12, 13], while the opposite may be the case for elevated dietary intakes of non-essential SFAs [14]. In this context, it has been shown that dietary supplementations with the n-3 PUFAs ALA or DHA can both diminish glucocorticoid secretion rates and related stress-induced anxiety- and depressive-like behaviors and cognitive impairments in rats [15, 16]. The dietary intake of SFAs is, by contrast, positively related to cortisol secretion rates in humans and the risk for mental disorders [17] and may impair behavioral and cognitive performances in rodents [18, 19]. These physiological and behavioral impacts are probably linked to the adverse effects of dietary PUFAs and SFAs on the availability, accumulation, and incorporation of long-chain PUFAs in the hippocampus and hypothalamus and neurotransmitter signaling in these brain areas [20, 21].

Varying fatty acid concentrations may further influence an individual’s social behavior, where aggressiveness has been shown to be especially related to lower n-3 PUFA levels in humans and animal models [22–24], but also to unbalanced dietary intakes of PUFAs and SFAs in general [25, 26]. Physiological and behavioral influences of dietary fatty acids may be of major importance for social interactions per se, since an individual’s social integrity could be directly affected by dietary fat types. Although specific dietary fatty acids, particularly n-3 PUFAs, have recently been shown to counteract social impairments in rats and mice [27, 28], rodent studies usually lack analyses in a fully-established social setup. This could provide an insight into the possible modulatory influences of dietary fatty acids in relation to the social environment and related social stressors. Studies in social-living macaques indicated that high-fat diets can modulate social behavior expression rates similarly under stable and unstable social environmental conditions, with animals maintained on diets high in PUFAs and SFAs showing less aggressive and more sociopositive behaviors [29, 30]. Diets high in ALA or LA can both diminish cortisol responses due to challenging social environmental conditions in guinea pigs [31]. These effects were related to increased locomotor activity, while social behaviors remained unaffected. Behavioral and physiological responses were stronger in males compared to females, but this was not modulated by the dietary fatty acids [31]. However, obvious sex differences in neurophysiological and behavioral functions [32, 33] and in the long-chain PUFA status [34] could possibly contribute to different effects of dietary fatty acids in male and female individuals.

The present study aimed to extend the findings on the modulatory influences of dietary fatty acids by comparing the effects of PUFAs and SFAs on saliva cortisol concentrations and social behaviors in male and female domestic guinea pigs (*Cavia aperea f. porcellus*) under different social conditions. Guinea pigs represent an adequate mammalian model species in this context, as these animals are highly social [35] and their physiological and behavioral stress responses to their social environment are well-studied [36–38]. Studying the effects of dietary PUFAs and SFAs in relation to the existing knowledge regarding social environmental influences in guinea pigs may therefore help to understand how these nutrients can affect social-living in male and female individuals and possibly shape social systems.

## Methods

### Animals

All domestic guinea pigs (30 males and 30 females) used for this study were bred at the Department of Behavioural Biology at the University of Vienna and originated from an established heterogeneous and multi-colored stock of animals at the department. All animals were adult and at the same developmental level, sexually intact, and accustomed to daily contact with humans. Individual identification was possible due to natural fur marks. Three months prior to the feeding experiments, animals were randomly assigned to single-sexed social groups of ten individuals (three male and three female groups) to enable an establishment of social relationships among the group members. Each group of animals was housed in enclosures of 3.2 m^2^. Each enclosure was environmentally enriched with shelters and the floor was covered with bedding material. The daily provided food consisted of ad libitum guinea pig chow (ssniff V2233, ssniff Spezialdiäten GmbH, Soest, Germany) and 50 g of hay per group. Water was available ad libitum in several drinking bottles. Animals were maintained on a light-dark cycle of 12/12 h (lights on at 07:00 a.m.) and at 20 ± 2 °C.

### Dietary regimes

After the three months of group formation, each male and female group was randomly assigned to one of three different dietary regimes, resulting in a 3 × 2 factorial experimental design of diet and sex. While the daily provided guinea pig chow remained the same for each of the previously established single-sexed groups, males and females on the same diet were additionally supplemented with either walnut oil (high in PUFAs) or coconut fat (high in SFAs) (see Table 1 for fatty acid composition of walnut oil and coconut fat). A control group was supplemented with pure water. For each animal, 3 ml of walnut oil, previously liquefied coconut fat (warmed at 30 °C), or pure water per kg body mass were administered orally using 1-ml syringes. The applied body mass-based supplementation procedure corresponds to previous studies in rats and guinea pigs where the oral administration of pure fatty acids or natural high-fat sources have successfully been proved to affect the fatty acid status in relation to physiological and behavioral changes [16, 39, 40]. A possible imbalance in the total energy intake among the dietary regimes was counterbalanced by ad libitum feeding of guinea pig chow.

**Table 1:** Fatty acid composition (% on total fatty acids) of walnut oil and coconut fat

Fatty acid	Walnut oil	Coconut fat
	(high-PUFA diet)	(high-SFA diet)
C 12:0	n.d.	49.27
C 14:0	n.d.	22.53
C 16:0	6.55	11.82
C 18:0	2.76	4.6
C 18:1 n-9	14.16	6.71
C 18:1 n-7	1.28	0.53
C 18:2 n-6	63.14	3.73
C 18:3 n-6	0.4	0.26
C 18:3 n-3	11.03	<0.01
total MUFAs	15.44	7.39
total PUFAs	75.25	4.22
total SFAs	9.31	88.4
n-6 : n-3 ratio	5.76	>38.85
PUFA : SFA ratio	8.08	0.05

Percentages of single fatty acids are based on gas chromatography analyses. *n.d*. not detectable

### Feeding experiments

The feeding experiments started with 60 days of group housing, where all animals remained in their previously established single-sexed groups throughout. Each day at 09:30 a.m., animals were weighed on a standard kitchen balance (accuracy: ± 1 g) and supplemented orally with walnut oil (PUFA group), coconut fat (SFA group), or pure water (control group), dependent on their body mass and the dietary regime the animals were assigned to. The whole procedure of weighing and supplementation lasted no longer than 1 min per animal. All shelters were removed from the enclosures during weighing and supplementing all individuals, which took approximately 60 min. The animals were therefore habituated to this situation, which was necessary for the following experimental steps, namely to provide visibility for video recordings.

On the last three consecutive days of group housing, all shelters were removed from the enclosures at 09:00 a.m. and each single-sexed group was video recorded for 30 min with cameras located directly above the enclosures and fixed at the ceiling. These video recordings were analyzed regarding the activity and social behavior expression rates of single individuals within their established social groups. At 09:30 a.m., directly after the daily video recordings, saliva samples were collected from all animals to analyze saliva cortisol concentrations as an indicator of the HPA-axis activity [38]. The chosen time of day for the experiments and saliva sample collections corresponds to highest activity rates and the cortisol peak across the diurnal rhythm in guinea pigs [41], where a maximum HPA-axis reactivity can be expected [42]. After day 60 of the group housing phase, blood samples were collected to analyze plasma fatty acids as an indicator of the fatty acid uptake from the diet and the general fatty acid status [43].

After the group housing phase and blood sample collections, all animals were subjected to 3-day social confrontations with animals of all sexes and dietary regimes. Social confrontations have previously been shown to constitute a highly stressful situation for guinea pigs, as animals are confronted with unfamiliar conspecifics without any established social relationships between them [31, 44]. On the day before the social confrontations started, animals were weighed and saliva samples were collected to analyze saliva cortisol levels. These body mass and saliva cortisol measurements served as baseline values in relation to the measurements during social confrontations in order to determine physiological influences of this new social environmental condition. For social confrontations, one male and one female of each dietary regime (in total three males and three females to form a group of six) were randomly chosen and transferred from their single-sexed groups to a square arena (1.44 m^2^), built of fiberboard, located on the floor in an adjacent room. All animals of the experiment were tested simultaneously in ten parallel setups. Directly after the transfer of the animals to the arenas at 09:00 a.m., as well as after 24 h and after 48 h, videos were recorded for 30 min via cameras located directly above the arenas and fixed at the ceiling. The video recordings served for an analysis of the animals’ behavior in this setup. After the video recordings, saliva samples were collected of each animal to analyze saliva cortisol levels and the body mass-based dietary fatty acid supplementation was carried out. Afterwards, all animals were relocated to their single-sexed groups. Guinea pig chow and water were available ad libitum throughout the social confrontations.

### Sample collection

Saliva samples were collected by inserting standard cotton buds into the animal’s mouth and gently collecting saliva from inside the cheeks for approximately 1 min. After centrifugation (14,000 rpm, 17,968 × g, 10 min), saliva was stored at −20 °C until further analysis. Blood samples were collected via prominent ear veins. Approximately 300 μl of blood was collected in heparinized micropipettes and centrifuged (14,000 rpm, 17,968 × g, 10 min) for plasma separation. Plasma was stored at −20 °C until further analyses. For further details regarding sample collection see [38].

### Hormone analyses

Cortisol concentrations in saliva were analyzed by biotin-strepdavidin enzyme-linked immunoassays [45, 46]. After thawing, saliva samples were diluted 1:50 and measured directly in 10-μl inputs using a cortisol-specific antibody. For cross-reactions of the used antibodies with relevant steroids see [46]. Hormone analyses were run in duplicates. Intra- and interassay coefficients of variance were 11.15 and 3.28 %.

### Plasma fatty acid analyses

Determination of free plasma fatty acids was carried out using gas chromatography, following previously established protocols [31, 47]. Fatty acids in 35 μl plasma were transesterificated by adding 1 ml methanolic NaOH, containing butylated hydroxytoluene. Samples were boiled for 5 min at 100 °C and cooled on ice for 10 min. 1 ml boron-trifluoride was added to obtain fatty acid methyl esters (FAMES) and the samples were shortly vortexed and again boiled and cooled on ice as described above. FAMES were extracted by adding 500 μl hexane four times, including 5 min of shaking the samples (700 rpm) in between, evaporated at 40 °C under nitrogen, and redissolved in hexane. FAMES were separated by an Rtx-2330 30 m × 0.25 mm × 0.20 μm silica column using an Auto-System-Gaschromatograph (Perkin Elmer, USA) with flame ionization detector. One microliter of prepared samples was injected under a 1:25 split at 250 °C and detected at 275 °C; helium was used as carrier gas. Identification of fatty acids was done by a 37 component FAME Mix Standard (Supelco, Bellafonte, USA). TotalChrome Workstation 6.3.0 (PE Nelson, Perkin Elmer, USA) was used for peak integration. Single fatty acids are expressed as percentage of total plasma fatty acids.

### Behavioral analyses

Videos were analyzed using the Observer XT 10 software (Version 10.5.572, Noldus, Wageningen, the Netherlands). Social behaviors as well as locomotor activity were recorded for each animal by applying a combined continuous and all occurrence sampling method [48]. Social behaviors were measured in frequencies, locomotion in durations. Behaviors and their categorization mainly followed the definitions by Rood [35]: (1) Locomotion: walking, running, jumping. (2) Socio-positive behaviors: social grooming, nose-nose contact, naso-anal sniffing. (3) Agonistic behaviors: chase, bite, fight, head-thrust, stand-threat, and kick-back.

The social rank was determined for each animal during group housing and separately for males and females during social confrontations to gain an insight into the social hierarchies. For this purpose, the Coulon Index was calculated by the proportion of won agonistic interactions on all agonistic interactions an individual was involved in [49]. Following previous approaches [50], the winner of an agonistic interaction is reliably indicated by the opponents retreat. A dyadic interaction was therefore defined as won, if the other individual retreated. During group housing, all six single-sexed groups contained the same number of animals and stayed together throughout the 60-day group housing period. Mathematically, each won agonistic interaction had to be lost by another individual in the same group and therefore the mean Coulon index within a single-sexed group was always 0.5 (= same numbers for total won and lost agonistic interactions). The absolute difference of the Coulon index of each animal from the mean Coulon index of 0.5 was further calculated. This resulted in higher values for individuals with very high and/or low Coulon indices and lower values for individuals with moderate Coulon indices around the mean. These measurements served as indicators of the Coulon index variability within the different single-sexed groups and the hierarchical structures. For social confrontations, the Coulon index was separately calculated for males and females, as both sexes show independent social hierarchies [51].

### Initial conditions

After the 3 months of group formation, the initial conditions, including body mass, age, saliva cortisol levels, and behavioral measurements were compared between each single-sexed group to control for possible differences in these variables in advance to the feeding experiments. Based on two-way analyses of variance using the statistical software package R 3.2.2 [52], no differences were found in body mass (*F*_5,54_ = 0.746, *p* = 0.593; mean body mass: 805 ± 20 gram) and age (*F*_5,54_ = 0.566, *p* = 0.726; mean age: 21.1 ± 1.2 months), but in saliva cortisol levels (*F*_5,52_ = 2.621, *p* = 0.035), which were generally higher in females compared to males (*F*_1,52_ = 7.849, *p* = 0.007; males: 6.26 ± 1.25 ng/ml, females: 42.81 ± 16.76 ng/ml). Further on, no differences were detected in locomotor activity (*F*_5,57_ = 0.726, *p* = 0.607; mean locomotion: 207.1 ± 12.6 sec/30 min) and sociopositive behaviors (*F*_5,55_ = 1.309, *p* = 0.274; mean sociopositive behaviors: 14.7 ± 1.8 /30 min), while a difference in agonistic behaviors (*F*_5,57_ = 2.448, *p* = 0.044) was caused by higher levels in males compared to females (males: 21.6 ± 1.8 /30 min; females: 12.6 ± 1.9 /30 min).

### Statistical analyses

Statistical analyses were carried out using the statistical software package R version 3.2.2 [52] and the implemented libraries “nlme” [53], for performing linear mixed effects models (LMEs), and “phia” [54], for post-hoc interaction analyses of significant interaction effects. Measurements were controlled for outliers beforehand using library “outliers” [55]. LMEs were mainly used for analyzing the data as these models allow adequate statistical corrections for repeated measurements and definitions of the variance structures.

As group housing and social confrontations represented two different social setups and the preconditions for each animal were highly divergent for both conditions, behavioral and physiological parameters were analyzed separately for these two social environments by applying condition-specific LMEs. Behaviors (locomotion, sociopositive behaviors, agonistic behaviors), the Coulon index variability, saliva cortisol concentrations, and body mass during group housing were analyzed by applying LMEs, including “diet” (control, PUFA, SFA), “sex” (males, females), and their interaction as fixed effects and individuals as random effects to correct for repeated measurements across the 3 days of group housing. The Coulon index for social confrontations was calculated and analyzed separately among males and females by applying LMEs including “diet” (control, PUFA, SFA), the Coulon index of group housing (Coulon index GH), and the mean Coulon index of group housing for all animals of the same sex within the same arena during social confrontation (Coulon index GHothers), and their interactions. Individuals within arena were included as random effects to correct for repeated measurements and the individual compositions of the arenas during social confrontations. As the former Coulon indices of group housing had no significant effects on the Coulon indices during social confrontations (see result section), a statistical adjustment to the Coulon index during social confrontations was not necessary. LMEs for behavioral and physiological parameters during social confrontations were therefore calculated including “diet” (control, PUFA, SFA), “sex” (males, females), and their interaction as fixed effects and individuals within arena as random effects. To control for possible influences of the group housing condition on the performances during social confrontations, each LME was also calculated including the respective parameter measured during group housing as a covariate, and based on the Akaike information criterion (AIC), compared to the respective model without this parameter. As the group housing parameter did not improve the models for social confrontations, group housing conditions therefore showing no effect on social confrontations, and due to the principle of parsimony, the initial models were chosen.

To analyze physiological responses to social confrontations, LMEs for saliva cortisol levels and body mass were applied, including “diet” (control, PUFA, SFA), “sex” (males, females), “time” (“before” and “during” social confrontations), and their interactions as fixed effects, and individuals as random effects to correct for repeated measurements. Plasma fatty acids determined at the end of group housing were analyzed by applying two-way ANOVAs, including “diet” (control, PUFA, SFA), “sex” (males, females), and their interaction as predictor variables.

Starting with full models, which contained all above-mentioned main effects and interactions, models were fitted based on the AIC. In case of significant interaction effects of “diet” and “sex”, post-hoc interaction analyses were carried out regarding sex differences within each diet group and differences between the diet groups within the sexes, respectively. All post-hoc analyses were Bonferroni corrected at the relevant level of analysis. Linearity, normality, and homoscedasticity of each model’s residuals were checked visually by plotting model diagnostics and by performing Shapiro-Wilk normality tests and Levene’s test for homogeneity of variance. Some of the response variables had to be transformed by applying the natural logarithm or the second or third root, but untransformed data was used for visualization of the results. Model statistics are based on type three sum of squares. The level of significance was set at *p* ≤ 0.05. All results are expressed as means ± standard errors.

## Results

### Plasma fatty acids

The dietary treatments resulted in significantly different plasma fatty acid patterns among males and females (Table 2). Corresponding to the dietary treatments, total plasma SFAs were in general highest in animals fed on the SFA-high diet, with SFA males showing higher levels than SFA females. Total plasma n-3 and n-6 fatty acids were highest in animals fed on the high-PUFA diet, with PUFA males showing lower n-3 levels than PUFA females. Total PUFAs and n-6 fatty acids, however, did not differ between the sexes. Also n-9 fatty acids and total MUFAs did not differ between the sexes, but were highest in control animals in general. The plasma n-6:n-3 ratio did not differ among males, but was significantly higher in SFA females compared to the other females and also higher in control males compared to control females.

**Table 2:** Plasma fatty acids for guinea pigs maintained on control, high-PUFA, or high-SFA diets

Fatty acid	Sex	Diet		
		Control	PUFA	SFA
C 12:0	Male	0.06 ± 0.01 ^a^	0.09 ± 0.00 ^a^	2.95 ± 0.68 ^b #^
	Female	0.06 ± 0.02 ^a^	0.07 ± 0.02 ^a^	1.27 ± 0.36 ^b #^
C 14:0	Male	0.50 ± 0.05 ^a^	0.49 ± 0.04 ^a^	5.72 ± 0.48 ^b #^
	Female	0.93 ± 0.08 ^a^	0.59 ± 0.04 ^a^	4.64 ± 0.61 ^b #^
C 16:0	Male	14.51 ± 0.60 ^ab #^	13.17 ± 0.46 ^a^	15.58 ± 0.29 ^b^
	Female	17.12 ± 0.23 ^a#^	12.70 ± 0.39 ^b^	16.80 ± 0.49 ^a^
C 18:0	Male	12.69 ± 0.30 ^a #^	9.15 ± 0.28 ^b #^	10.09 ± 0.31 ^b^
	Female	10.09 ± 0.52 ^a #^	6.84 ± 0.41 ^b #^	9.05 ± 0.40 ^a^
C 18.1 n-9	Male	14.85 ± 1.44 ^a^	11.75 ± 0.46 ^b^	12.03 ± 0.23 ^b^
	Female	15.53 ± 0.46 ^a^	12.85 ± 0.70 ^b^	12.49 ± 0.59 ^b^
C 18:2 n-6	Male	42.91 ± 1.51 ^a^	48.81 ± 0.98 ^b^	39.95 ± 0.90 ^a^
	Female	39.77 ± 1.00 ^a^	49.13 ± 0.86 ^b^	41.73 ± 0.60 ^a^
C 18:3 n-3	Male	5.33 ± 0.33 ^a^	6.46 ± 0.14 ^b #^	4.79 ± 0.20 ^a^
	Female	6.02 ± 0.22 ^a^	7.71 ± 0.14 ^b #^	4.47 ± 0.36 ^c^
Total n-9	Male	15.52 ± 1.50 ^a^	14.31 ± 0.30 ^ab^	12.60 ± 0.29 ^b^
	Female	16.21 ± 0.47 ^a^	15.42 ± 0.46 ^ab^	13.13 ± 0.58 ^b^
Total n-6	Male	46.38 ± 1.70 ^a^	51.22 ± 0.99 ^b^	42.47 ± 0.94 ^c^
	Female	43.09 ± 0.93 ^a^	51.41 ± 0.90 ^b^	44.62 ± 0.67 ^a^
Total n-3	Male	5.77 ± 0.32 ^a^	6.82 ± 0.13 ^b #^	5.14 ± 0.20 ^a^
	Female	6.64 ± 0.24 ^a^	8.12 ± 0.45 ^b #^	4.95 ± 0.36 ^c^
Total SFA	Male	30.70 ± 0.50 ^a^	26.28 ± 0.88 ^b^	38.13 ± 1.14 ^c #^
	Female	31.67 ± 0.74 ^a^	23.74 ± 0.91 ^b^	35.31 ± 0.81 ^c #^
Total MUFA	Male	17.06 ± 1.45 ^a^	15.59 ± 0.33 ^ab^	14.14 ± 0.32 ^b^
	Female	18.51 ± 0.50 ^a^	16.66 ± 0.49 ^ab^	15.03 ± 0.63 ^b^
Total PUFA	Male	52.24 ± 1.67 ^a^	58.12 ± 0.96 ^b^	47.73 ± 0.94 ^c^
	Female	49.82 ± 1.08 ^a^	59.60 ± 0.89 ^b^	49.66 ± 0.55 ^a^
n-6 : n-3 ratio	Male	8.33 ± 0.72 ^#^	7.55 ± 0.24	8.38 ± 0.38
	Female	6.55 ± 0.22 ^a #^	6.57 ± 0.51 ^a^	9.59 ± 0.88 ^b^

Values for single types of fatty acids represent percentages on total fatty acids. Different superscript letters indicate significant differences between the dietary treatments within the sexes (*p* ≤ 0.05). ^#^Significant difference between the sexes within the dietary treatments (*p* ≤ 0.05)

**Fig. 1:**
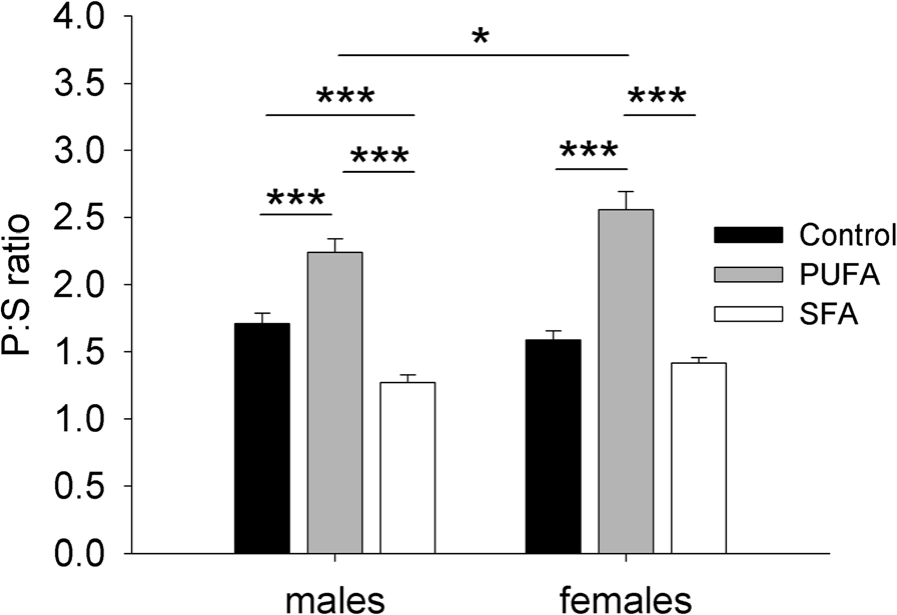
Plasma polyunsaturated:saturated fatty acid (P:S) ratio for guinea pigs maintained on control, high-PUFA, or high-SFA diets. Pairwise post-hoc comparisons were carried out between the sexes within each dietary treatment and between the dietary treatments within the sexes. *p ≤ 0.05, ***p ≤ 0.001

Regarding the plasma PUFA:SFA (P:S) ratio, as a marker for the overall PUFA- and SFA-status, a significant interaction of diet and sex was found (diet: *F*_2,54_ = 51.563, *p* < 0.001; sex: *F*_1,54_ = 1.042, *p* = 0.312; diet:sex: *F*_2,54_ = 3.326, *p* = 0.043) (Fig. 1). A sex difference in the P:S ratio was found in relation to the high-PUFA diet, with males exhibiting a lower ratio than females (*F*_1,54_ = 6.725, *p* = 0.037). No sex differences in the P:S ratio were found in relation to the other diets (control: *F*_1,54_ = 1.042, *p* = 0.936; SFA: *F*_1,54_ = 1.476, *p* = 0.689). Comparing the dietary treatments within the sexes revealed that PUFA males showed the highest, SFA males the lowest, and control males an intermediate P:S ratio (control-PUFA: *F*_1,54_ = 19.209, *p* < 0.001; control-SFA: *F*_1,54_ = 13.380, *p* < 0.001; PUFA-SFA: *F*_1,54_ = 64.651, *p* < 0.001). Also PUFA females showed the highest P:S ratio (control-PUFA: *F*_1,54_ = 63.946, *p* < 0.001; PUFA-SFA: *F*_1,54_ = 88.719, *p* < 0.001), with no differences between control and SFA females (*F*_1,54_ = 2.023, *p* = 0.964).

### Saliva cortisol concentrations

**Fig. 2:**
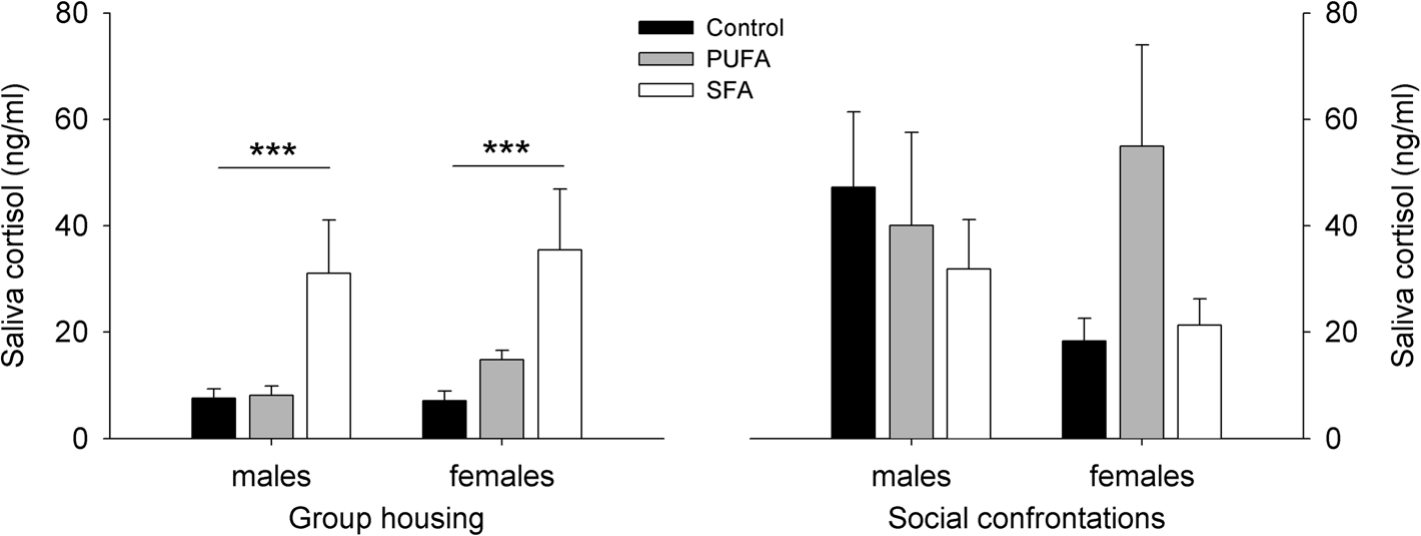
Saliva cortisol concentrations for guinea pigs maintained on control, high-PUFA, or high-SFA diets. Analyses were carried out separately for group housing and social confrontations. Pairwise post-hoc comparisons for group housing and social confrontations were carried out between the sexes within each dietary treatment and between the dietary treatments within the sexes. ***p ≤ 0.001 comparing control and SFA diets (males + females) in general

The dietary treatments had a significant effect on saliva cortisol levels during group housing, with no differences between the sexes (diet: *F*_2,55_ = 7.935, *p* < 0.001; sex: *F*_2,53_ = 2.101, *p* = 0.153) (Fig. 2). The interaction effect of diet and sex was removed beforehand (*F*_2,53_ = 1.222, *p* = 0.303). Individuals maintained on the SFA diet showed significantly elevated saliva cortisol levels compared to the control diet (*χ*^*2*^ = 15.413, *p* < 0.001), while no significant effects were detected for the PUFA diet (control-PUFA: *χ*^*2*^ = 5.179, *p* = 0.069; PUFA-SFA: *χ*^*2*^ = 4.693, *p* = 0.091).

**Fig. 3:**
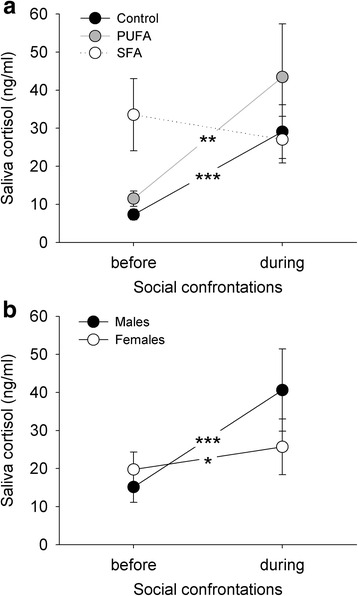
Change in saliva cortisol concentrations for guinea pigs due to social confrontations. **a** Effect of dietary treatments (control, PUFA, SFA), combining both sexes. **b** Effect of sex, combining all dietary treatments. *p ≤ 0.05, **p ≤ 0.01, ***p ≤ 0.001 comparing both sampling points in time within the dietary treatments. Comparison of time courses: control-PUFA: n.s., control-SFA: *p* ≤ 0.001, PUFA-SFA: *p* ≤ 0.05; males-females: *p* = 0.051

Social confrontations had a significant impact on saliva cortisol levels in relation to the dietary treatments and an almost significant impact in relation to the sexes (diet: *F*_2,53_ = 8.514, *p* < 0.001; sex: *F*_1,53_ < 0.001; *p* = 0.982; time: *F*_1,55_ = 23.437, *p* < 0.001; diet:sex: *F*_2,53_ = 1.980, *p* = 0.148; diet:time: *F*_2,55_ = 5.106, *p* = 0.009; sex:time: *F*_1,55_ = 3.984, *p* = 0.051) (Fig. 3). Animals maintained on the control and PUFA diets showed an increase in saliva cortisol concentrations due to the onset of social confrontations, while SFA animals showed no such change in cortisol levels (control: *χ*^*2*^ = 43.745, *p* < 0.001; PUFA: *χ*^*2*^ = 9.968, *p* = 0.002; SFA: *χ*^*2*^ = 0.004, *p* = 0.948). This increase in saliva cortisol in control and PUFA animals was significantly different to SFA animals (control-PUFA: *χ*^*2*^ = 0.701, *p* = 0.402; control-SFA: *χ*^*2*^ = 11.157, *p* < 0.001; PUFA-SFA: *χ*^*2*^ = 4.305, *p* = 0.038). The general increase in saliva cortisol levels in males was almost significantly higher compared to females (males: *χ*^*2*^ = 18.574, *p* < 0.001; females: *χ*^*2*^ = 4.751, *p* = 0.029).

No differences in saliva cortisol concentrations were detected between the dietary treatments or sexes during the 3 days of social confrontations, resulting in an elimination of all predictors during model fitting (full model: diet: *F*_2,44_ = 1.011; *p* = 0.372; sex: *F*_1,44_ = 0.536, *p* = 0.468; diet:sex: *F*_2,44_ = 0.580, *p* = 0.564) (Fig. 2).

### Body mass

The animals’ body mass during group housing remained unaffected by the dietary treatments and did also not differ between the sexes (full model: diet: *F*_2,55_ = 1.940, *p* = 0.153; sex: *F*_1,55_ = 1.657, *p* = 0.203; diet:sex: *F*_2,55_ = 1.361, *p* = 0.265). The overall mean body mass during group housing was 889.2 ± 14.8 g.

A general loss in body mass was detected due to social confrontations, which did not differ significantly between the sexes (sex: *F*_1,59_ = 0.009; *p* = 0.924; time: *F*_1,57_ = 55.380, *p* < 0.001; sex:time: *F*_1,57_ = 3.608, *p* = 0.063), and also all diet-related effects remained non-significant and were therefore removed during model fitting beforehand (diet: *F*_2,55_ = 1.843, *p* = 0.168; diet:sex: *F*_2,55_ = 1.292, *p* = 0.283; diet:time: *F*_2,53_ = 1.254, *p* = 0.294; diet:sex:time: *F*_2,53_ = 1.034, *p* = 0.363).

Furthermore, no differences in body mass during social confrontations were detected (full model: diet: *F*_2,44_ = 1.971, *p* = 0.152; sex: *F*_1,44_ = 0.024, *p* = 0.878; diet:sex: *F*_2,44_ = 0.701, *p* = 0.501). The overall mean body mass during social confrontations was 857.6 ± 17.1 g.

### Coulon indices

The Coulon index variability during group housing did not differ between the diets and/or sexes (full model: diet: *F*_2,55_ = 0.937, *p* = 0.398; sex: *F*_1,55_ = 0.160, *p* = 0.691; diet:sex: *F*_2,55_ = 0.590, *p* = 0.558). The mean Coulon index variability for the single-sexed groups was 0.285 ± 0.017.

**Fig. 4:**
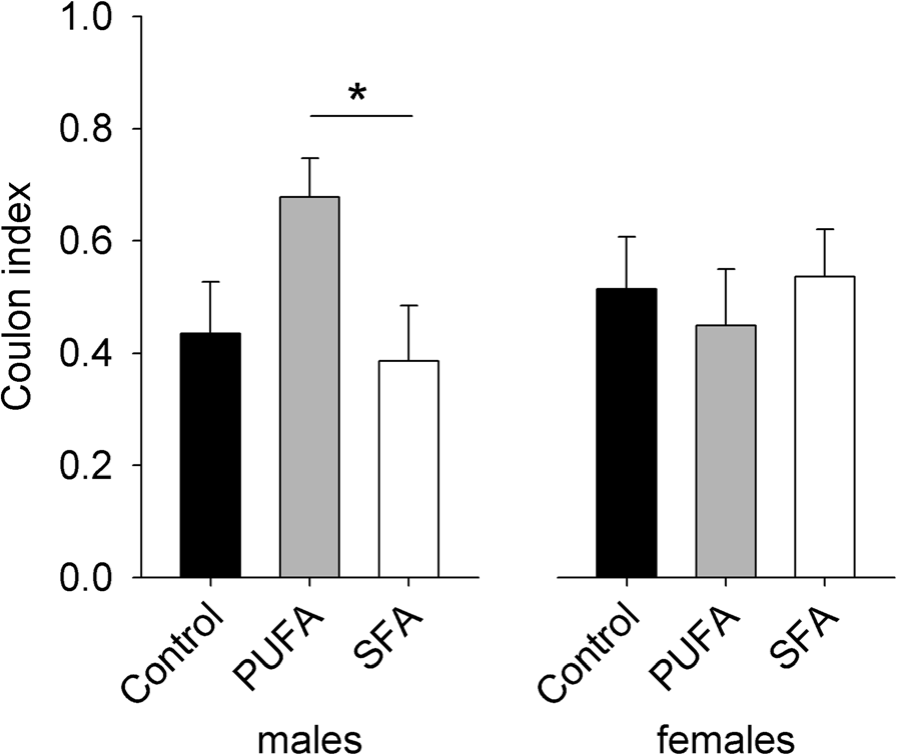
Coulon index for guinea pigs maintained on control, high-PUFA, or high-SFA diets during social confrontations. Analyses and pairwise post-hoc comparisons were carried out separately for the sexes. *p ≤ 0.05

The Coulon index for males during social confrontations was significantly affected by the dietary treatment, while an effect of the former Coulon index in the established single-sexed groups during group housing remained non-significant (diet: *F*_2,17_ = 4.027; *p* = 0.037; Coulon index GH: *F*_1,17_ = 4.040, *p* = 0.061) (Fig. 4). Males maintained on the PUFA diet showed the highest Coulon indices across the social confrontations, which was significantly different to SFA males and nearly to control males (control-PUFA: *χ*^*2*^ = 5.482, *p* = 0.058; PUFA-SFA: *χ*^*2*^ = 7.052, *p* = 0.024), while control and SFA males did not differ in their Coulon indices (control-SFA: *χ*^*2*^ = 0.095, *p* = 1.000). All other predictor main effects and interactions, including the Coulon indices of the other individuals within a social confrontation setup, were not significant and therefore removed from the model beforehand due to model fitting based on the AIC (Coulon index GHothers: *F*_1,8_ = 0.394, *p* = 0.548; diet:Coulon index GH: *F*_2,13_ = 0.615, *p* = 0.556; diet:Coulon index GHothers: *F*_2,13_ = 0.007, *p* = 0.993).

The Coulon index for females during social confrontations remained unaffected by the dietary treatment and also by the previous Coulon indices (full model: diet: *F*_2,12_ = 1.132, *p* = 0.354; Coulon index GH: *F*_1,12_ = 2.072, *p* = 0.176; Coulon index GHothers: *F*_1,8_ = 0.179, *p* = 0.684; diet: Coulon index GH: *F*_2,12_ = 0.067, *p* = 0.936; diet:Coulon index GHothers: *F*_2,12_ = 0.626, *p* = 0.551) (Fig. 4).

### Locomotion

**Fig. 5:**
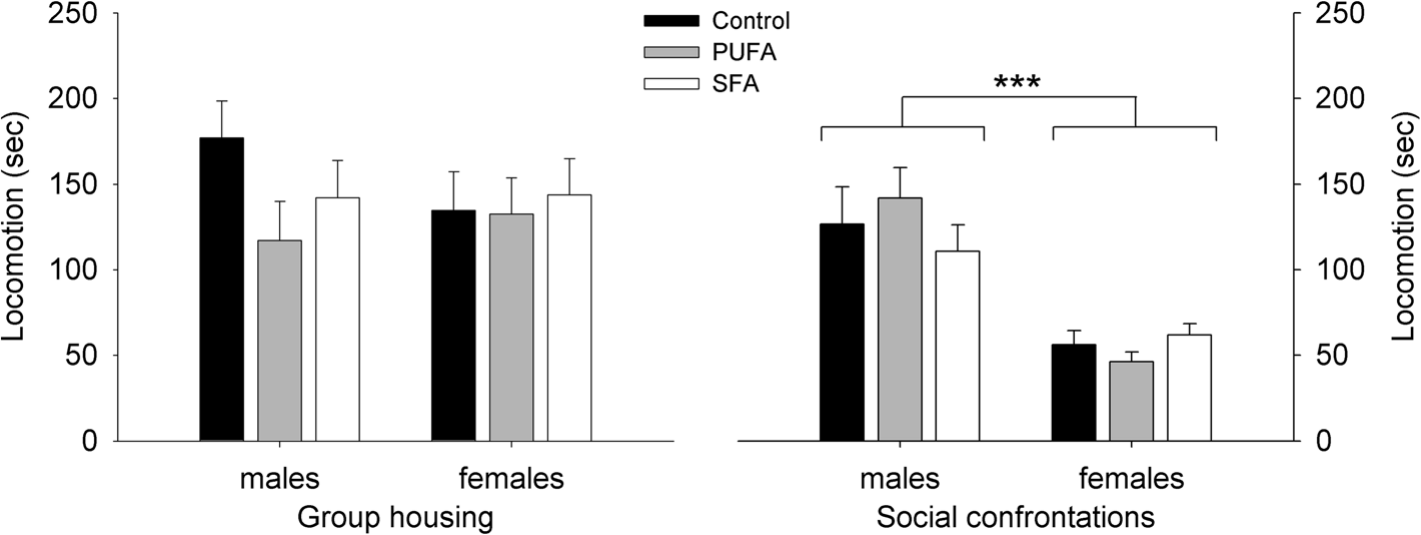
Locomotor activity for guinea pigs maintained on control, high-PUFA, or high-SFA diets. Analyses were carried out separately for group housing and social confrontations. Pairwise post-hoc comparisons for group housing and social confrontations were carried out between the sexes within each dietary treatment and between the dietary treatments within the sexes. ***p ≤ 0.001 comparing all males and females

Locomotor activity during group housing was not affected by the dietary treatments and did also not differ between the sexes, resulting in an elimination of all predictors during model fitting (full model: diet: *F*_2,55_ = 0.087, *p* = 0.917; sex: *F*_1,55_ = 0.522, *p* = 0.473; diet:sex: *F*_2,55_ = 0.640, *p* = 0.531) (Fig. 5).

During social confrontations, a general sex difference in locomotion was found (*F*_1,48_ = 33.849, *p* < 0.001), with males exceeding females (Fig. 5), but no diet-related effects were detected (full model: diet: *F*_2,44_ = 1.493, *p* = 0.236; sex: *F*_1,44_ = 8.081, *p* = 0.007; diet:sex: *F*_2,44_ = 1.809, *p* = 0.176).

### Sociopositive behaviors

**Fig. 6:**
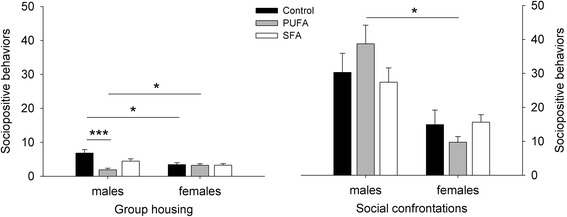
Sociopositive behavior for guinea pigs maintained on control, high-PUFA, or high-SFA diets. Analyses were carried out separately for group housing and social confrontations. Pairwise post-hoc comparisons for group housing and social confrontations were carried out between the sexes within each dietary treatment and between the dietary treatments within the sexes. *p ≤ 0.05, ***p ≤ 0.001

Regarding sociopositive behaviors during group housing, a significant interaction effect of diet and sex was detected (diet: *F*_2,55_ = 0.028, *p* = 0.972; sex: *F*_1,55_ = 5.552, *p* = 0.022; diet:sex: *F*_2,55_ = 5.764, *p* = 0.005) (Fig. 6). While control males showed more sociopositive behaviors than control females (*χ*^*2*^ = 5.741, *p* = 0.050), PUFA males showed less sociopositive behaviors than PUFA females (*χ*^*2*^ = 5.828, *p* = 0.047). No sex difference was detected in relation to the SFA diet (*χ*^*2*^ = 0.376, *p* = 1.000). Comparing the dietary treatments within the sexes revealed significantly less sociopositive behaviors in PUFA males compared to control males (control-PUFA: *χ*^*2*^ = 19.644, *p* < 0.001; control-SFA: *χ*^*2*^ = 3.393, *p* = 0.393; PUFA-SFA:*χ*^*2*^ = 6.418, *p* = 0.068), while no differences were detected among females (*χ*^*2*^ = 0.058, *p* = 1.000).

Also for social confrontations a significant interaction effect of diet and sex on sociopositive behaviors was detected (diet: *F*_2,44_ = 2.930, *p* = 0.064; sex:* F*_1,44_ = 2.385, *p* = 0.130; diet:sex: *F*_2,44_ = 3.379, *p* = 0.043) (Fig. 6). In contrast to the group housing condition, however, PUFA males showed significantly more sociopositive behaviors than PUFA females (*χ*^*2*^ = 5.828, *p* = 0.047), while no sex differences were detected in relation to the control and SFA diets (control: *χ*^*2*^ = 2.468, *p* = 0.349; SFA: *χ*^*2*^ = 2.924, *p* = 0.262). Comparing the dietary treatments within the sexes revealed no significant differences among males (*χ*^*2*^ = 3.008, *p* = 0.444) or females (*χ*^*2*^ = 6.067, *p* = 0.096).

### Agonistic behaviors

**Fig. 7:**
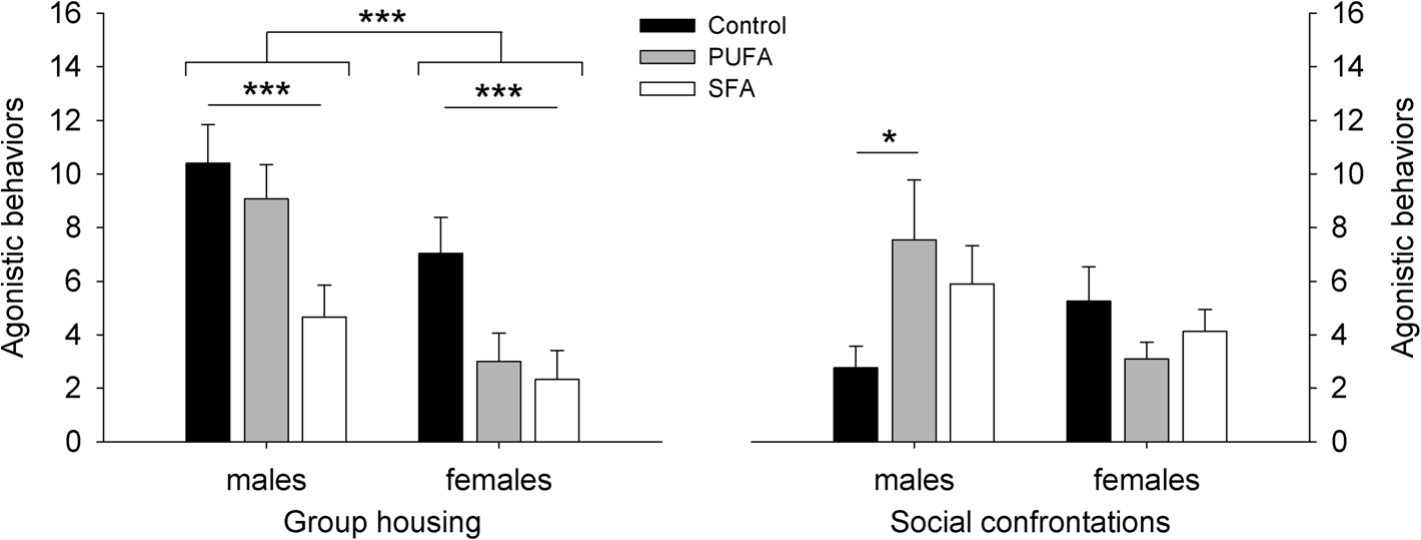
Agonistic behavior for guinea pigs maintained on control, high-PUFA, or high-SFA diets. Analyses were carried out separately for group housing and social confrontations. Pairwise post-hoc comparisons for group housing and social confrontations were carried out between the sexes within each dietary treatment and between the dietary treatments within the sexes. * *p* ≤ 0.05, *** *p* ≤ 0.001

Agonistic behaviors during group housing significantly differed between the dietary treatments and the sexes in general (diet: *F*_2,57_ = 7.540, *p* = 0.001; sex: *F*_1,57_ = 15.174, *p* < 0.001) (Fig. 7). Males generally displayed more agonistic behaviors than females. Additionally, the SFA diet resulted in decreased agonistic behavioral expression rates compared to the control diet (*χ*^*2*^ = 15.382, *p* < 0.001), while no effects were detected for the PUFA diet (control-PUFA: *χ*^*2*^ = 3.832, *p* = 0.151; PUFA-SFA: *χ*^*2*^ = 3.983, *p* = 0.138). The interaction effect of diet and sex was eliminated beforehand (*F*_2,55_ = 1.362, *p* = 0.265).

A significant interaction effect of diet and sex on agonistic behaviors was detected during social confrontations (diet: *F*_2,44_ = 0.850, *p* = 0.434; sex: *F*_1,44_ = 5.246, *p* = 0.027; diet:sex: *F*_2,44_ = 3.891, *p* = 0.028) (Fig. 7).While no sex differences were detected within the single dietary treatments (control: *χ*^*2*^ = 5.430, *p* = 0.059; PUFA: *χ*^*2*^ = 2.764, *p* = 0.289; SFA: *χ*^*2*^ = 0.002, *p* = 1.000), PUFA males showed significantly more agonistic behaviors compared to control males (control-PUFA: *χ*^*2*^ = 7.312, *p* = 0.041; control-SFA: *χ*^*2*^ = 3.130, *p* = 0.461; PUFA-SFA: *χ*^*2*^ = 0.874, *p* = 1.000). No differences were detected among females (*χ*^*2*^ = 1.760, *p* = 0.830).

## Discussion

This study was performed to investigate the effects of diets enriched in either PUFAs or SFAs on saliva cortisol concentrations and social behaviors in different social environments in male and female guinea pigs. The dietary fatty acids affected the plasma fatty acid status in a sex-specific manner and showed pronounced modulatory influences on social behavior expression rates and related basal saliva cortisol secretions and cortisol responses during social confrontations. Dietary fatty acids also highly affected the establishment of a social hierarchy during social confrontations, insofar as PUFA males became dominant. These findings in social-living guinea pigs show how dietary fatty acids can modulate physiological and behavioral functions and probably shape social societies and social hierarchies differently in male and female individuals.

Fatty acids in plasma are a major source of polyunsaturated fatty acids for neuromembrane phospholipids in the brain [43]. The measurements of plasma fatty acids in this study should therefore reliably reflect the general fatty acid status. Although the plasma P:S ratio was highest for the PUFA group, PUFA males showed lower plasma n-3 levels and P:S ratios compared to PUFA females. Recent findings suggest a sexual dimorphism regarding the accumulation and metabolism of n-3 fatty acids, resulting in a generally higher n-3 fatty acid status in females compared to males [34, 56]. These findings could definitely be confirmed by our study and possibly extended by the sex differences which were also found for plasma SFAs, with SFA males showing higher levels than SFA females. PUFAs and SFAs are differently involved in metabolic processes, since PUFAs seem to be much faster oxidized and SFAs are more likely to be stored in abdominal fat compartments [57, 58]. A sexual dimorphism in such metabolic processes may be linked to different incorporation rates of specific fatty acids in target tissues and could therefore elicit sexually different effects on neurophysiological functions and behavioral expression rates.

Maintaining socially housed guinea pigs on diets high in PUFAs or SFAs already showed different effects on basal saliva cortisol levels in this study. Elevated HPA-axis activities were found for males and females maintained on SFAs, as indicated by increased saliva cortisol levels during group housing. This effect could be related to a low availability of PUFAs for integrations into neuronal cell membranes, although adequate PUFA contents are required for optimal HPA-axis functions [59, 60]. Diets high in SFAs, however, have also been shown to increase plasma low-density-lipoprotein and total cholesterol levels in guinea pigs [61], which may, at least in humans, be primarily used for steroid hormone synthesis by the adrenal glands [62], possibly resulting in elevated glucocorticoid secretion rates. Based on the findings presented here, no sexual dimorphism can be assumed for these reactions. In case of chronically elevated glucocorticoid levels, this can dramatically impair an individual’s physiological, metabolic, and behavioral functions and may result in the development of pathologies [63]. Interestingly, dietary PUFAs did not affect saliva cortisol concentrations during group housing at all. Previous studies showed that PUFAs can diminish glucocorticoid secretions under basal conditions, without stimulations of the HPA-axis, in individually housed guinea pigs as well as in humans [40, 64]. Studies in socially housed rats, however, revealed diminishing effects of n-3 PUFAs under stressful conditions, such as individual subjections to restraint stress, while basal glucocorticoid levels remained unaffected [16, 65]. The social environment and a tight link of social behaviors to the HPA-axis activity and neurotransmitter signaling in the serotonergic and dopaminergic systems [1] may adversely modulate the influences of n-3 PUFAs on the HPA-axis, perhaps especially in rodents.

In addition to the excessive HPA-axis functions, SFA males and females showed significantly reduced agonistic behaviors compared to control animals. Elevated glucocorticoid concentrations can reduce testosterone levels, which can lead to a general decrease in aggressiveness [66]. The potential negative effects of dietary SFAs on the basal HPA-axis activity could have therefore also been beneficial in terms of decreased aggressiveness towards group members. As no differences were detected in other behaviors, the lower conflict rates in SFA animals can indeed be assumed to be related to the described hormonal effect, which primarily affects aggressiveness. PUFAs, however, seemed to negatively affect sociopositive behaviors in male individuals, as indicated by lower expression rates compared to control males and PUFA females. Due to the previously reported positive effects of n-3 PUFAs on brain-related behavioral functions [13], more sociopositive and less agonistic behaviors could have been expected in PUFA animals. However, a study in humans showed that aggressiveness may remain unaffected by dietary n-3 fatty acids under non-stressful conditions [67], while the effects of dietary fatty acids on aggressiveness in rodents were mainly investigated using the stressful resident-intruder paradigm [22, 68]. All animals in this study were housed in established social groups during the initial feeding phase, including general sex differences in social behaviors, which is probably caused by the general higher activity and conflict rate in males [31]. Dietary PUFAs may have perhaps not further modulated social behaviors at this basal condition where social interactions contribute to the maintenance of the established social system. It could therefore be possible that the positive effects of PUFAs on social behavior and HPA-axis activities only show up under stressful and stimulated conditions.

Compared to the group housing condition, social confrontations represented an unpredictable and highly stressful social environment. The animals’ stress responses to the social confrontations were indicated by increased saliva cortisol levels and decreased body masses in both sexes. Saliva cortisol increases in males were almost significantly higher compared to females, indicating that social confrontations are more challenging for male individuals [31], probably due to high levels of intrasexual competition [44, 69]. This was generally indicated by a higher locomotor activity in males compared to females. Although guinea pigs usually react with immobility to stressful situations [70], this could be diminished in order to establish social relationships [37]. Elevated cortisol concentrations during social confrontations may have further resulted in decreasing body masses in all animals. According to that, glucocorticoids play an important role for energy balance [71] and the body mass loss may indicate catabolic effects of glucocorticoids in order to provide energy due to the social stressor. A stress-related decrease in body mass can also be diminished by n-3 PUFAs [65], which was suggested to be related to a different metabolic rate that could perhaps be caused by the predominant oxidation of PUFAs for energy provision [58]. Such an effect on the body mass could not be confirmed for guinea pigs so far [31] and was also not found in the recent study. However, as glucocorticoids stimulate metabolic processes, a close relation to metabolic rates can be assumed [72], which may also be affected by PUFAs.

Adequate responses to challenging physiological and/or environmental conditions and a related increase in glucocorticoid concentrations are of major importance as they enable individuals to cope with the prevalent stressors [3, 5, 63]. Dietary n-3 and n-6 PUFAs can both diminish such cortisol responses to social confrontations in individually housed guinea pigs [31], indicating a lower stress response in these animals. This effect could not been shown for group-housed animals maintained on a high-PUFA diet in this study, as PUFA animals showed similar cortisol responses to social confrontations as control individuals. Social housing conditions can strongly modulate cortisol responses to challenging situations in guinea pigs [73, 74]. The transfer from one social environment to another and a related change in the individual social status may have elicited pronounced cortisol responses in order to provide enough energy during social confrontations in this study. This was perhaps inevitable to cope with the situation and was therefore not diminished by dietary PUFAs, but perhaps enabled adequate and energetically demanding behavioral responses, which finally resulted in higher dominant ranks at least in PUFA males. However, elevated saliva cortisol levels during group housing in SFA animals perhaps impaired HPA-axis responses to social confrontations in these animals. Dietary SFAs have previously been shown to result in a suppression of cortisol responses to ACTH-challenges (injection of adrenocorticotropic hormone) in pigs [75] and are related to lower cortisol variability in humans [76]. Unfavorable P:S ratios may in general negatively affect HPA-axis functions and the already increased basal cortisol levels detected during group housing could have additionally impaired adequate cortisol responses to unpredictable stressors, such as social confrontations. As inappropriate HPA-axis activities and responses can strongly impair behavioral performances [77, 78], the missing cortisol response in SFA animals may have also influenced the way these animals adapted behaviorally to the social confrontations in this study.

SFA males were seemingly not able to adjust their behavior to the challenging conditions of social confrontations adequately. Although their sociopositive and agonistic behavior expression rates were similar to those observed in PUFA males, significantly lower Coulon indices suggest that SFA males lost more agonistic interactions and became subdominant. Interestingly, also control males failed in their behavioral adaptations, because they showed less agonistic behaviors compared to PUFA males and nearly became subdominant too. Contrasting to the reported positive effects of PUFAs in relation to lowering aggressiveness [13, 22, 23], high levels of aggressiveness were found in PUFA males during social confrontations. However, aggressive behaviors may also be facilitated by acute stressors and glucocorticoids, which has previously been shown in rats [79]. If the agonistic response of PUFA males to the social confrontations was caused by the rise in saliva cortisol levels, this effect even might have been of major importance in terms of becoming dominant, which may further increase their access to resources and females [80]. Control and SFA males therefore probably behaved unfavorably during social confrontations, which might be related to a decreased HPA-axis response and unbalanced P:S ratios, respectively. Recent findings in mice and rats showed that dietary supplementations with the n-3 fatty acid DHA can counteract social behavioral impairments, which were caused by the challenging environmental and physiological conditions of food allergy and prenatal ethanol exposure [27, 28]. In relation to these findings, dietary PUFAs in general may also positively affect social behaviors under stressful social environmental conditions as found here. In contrast to males, the females’ Coulon indices and behavioral frequencies were similar among the different dietary treatments. In general, both male and female guinea pigs show social hierarchies within the respective sex [51], but this is definitely more pronounced in males than in females. Males are therefore probably more sensitive to environmental and physiological changes and may rather adjust their behavior to the prevalent conditions than females. This sex-specific effect would correspond to previous findings, where different PUFAs have been shown to positively affect spatial cognitive abilities in guinea pigs and locomotor activity in rats in a sex-specific manner [40, 81].

## Conclusions

This study highlights the importance of balanced dietary intakes of PUFAs and SFAs regarding cortisol secretion rates and behavioral expression rates in different social environments. Sex differences in the individual fatty acid status, following dietary supplementation with PUFAs and SFAs, and perhaps in the metabolization of these nutrients may have influenced the way males and females adapted physiologically and behaviorally to a changing social environment. These new findings on the positive effects of PUFAs on social interactions and the negative effects of SFAs on HPA-axis functions are of major importance for social-living animals and should be expanded to higher evolved mammals as well as human societies. Several civilization diseases, including obesity or cardiovascular diseases, and mood or behavioral disorders, which are linked to hormonal and behavioral dysfunctions, are caused by imbalanced dietary intakes of macronutrients, with emphasis on dietary fatty acids [10, 82, 83]. These physiological and behavioral impairments may profoundly impact on social-living, where individuals are regularly confronted with stressful situations and have to respond to these situations appropriately. Adequate dietary intakes of n-3 and n-6 PUFAs and SFAs play an important role in the prevention of brain- and metabolic-related diseases in relation to hormonal and behavioral dysfunctions and can therefore improve an individual’s health and social integrity. However, a limitation of the presented results on sex-specific influences of these nutrients may have been caused by metabolic sex differences in the investigated species per se, the single-sexed housing conditions, or even an interaction of both factors. Although the performed statistical analyses support sex-specific effects of dietary fatty acids and helped to control for the mentioned influences, they cannot be fully excluded and therefore further investigations are encouraged.

## Additional file

Additional file 1: Dataset supporting the results and conclusions of this article. Physiological and behavioral data collected during group housing and social confrontations. (XLS 76 kb)

## Abbreviations

AA: Arachidonic acid
AIC: Akaike information criterion
ALA: Alpha-linolenic acid
DHA: Docosahexaenoic acid
EPA: Eicosapentaenoic acid
FAMES: Fatty acid methyl esters
HPA-axis: Hypothalamic-pituitary-adrenal-axis
LA: Linoleic acid
LME: Linear mixed effects model
n-3: Omega-3
n-6: Omega-6
PUFA: Polyunsaturated fatty acid
SFA: Saturated fatty acid
